# First field study using *Strong*-LAMP for diagnosis of strongyloidiasis in Cubal, Angola

**DOI:** 10.1186/s13071-023-06009-3

**Published:** 2023-10-31

**Authors:** Beatriz Crego-Vicente, Begoña Febrer-Sendra, Arlette Nindia, Agostinho Pessela, Sandra Aixut, Joan Martínez-Campreciós, Alejandro Mediavilla, Aroa Silgado, Elena Sulleiro, Begoña Treviño, Israel Molina, Antonio Muro, Fernando Salvador, Pedro Fernández-Soto

**Affiliations:** 1 Infectious and Tropical Diseases Research Group (e-INTRO), Biomedical Research Institute of Salamanca-Research Centre for Tropical Diseases at the University of Salamanca (IBSAL-CIETUS), Faculty of Pharmacy, University of Salamanca, Salamanca, Spain; 2 Hospital Nossa Senhora da Paz, Cubal, Angola; 3 Centro de Investigación Biomédica en Red de Enfermedades Infecciosas (CIBERINFEC), Instituto de Salud Carlos III, Madrid, Spain; 4 Microbiology Department, Vall d’Hebron University Hospital, PROSICS, Barcelona, Spain; 5 International Health Unit Vall d’Hebron-Drassanes, Infectious Diseases Department, Vall d’Hebron University Hospital, PROSICS, Barcelona, Spain

**Keywords:** Strongyloidiasis, Field study, Molecular screening, Loop-mediated isothermal amplification (LAMP), *Strong*-LAMP, Direct saline microscopy, Baermann, Stool samples, Cubal, Angola

## Abstract

**Background:**

*Strongyloides stercoralis* infection is a common neglected tropical disease distributed worldwide, mainly in tropical and subtropical climates. The impact of *S. stercoralis* infections on human health ranges from mild asymptomatic infections to chronic strongyloidiasis unnoticeable until the host is immunosuppressed. In severe strongyloidiasis, a syndrome of hyperinfection and larval dissemination to various organs can occur with high mortality rates. The diagnosis of strongyloidiasis is challenging because of the absence of a single standard reference test with high sensitivity and specificity, which also makes it difficult to estimate the accuracy of other diagnostic tests. This study aimed to evaluate, for the first time, the use of an easy-to-perform loop-mediated isothermal amplification (LAMP) colorimetric assay (named *Strong*-LAMP) for the molecular screening of strongyloidiasis in stool samples from patients in a low-resource endemic area in Cubal, Angola. To compare different LAMP application scenarios, the performance of the *Strong*-LAMP under field conditions in Angola was reassessed in a well-equipped reference laboratory in Spain and compared with a quantitative polymerase chain reaction (qPCR) method.

**Methods:**

A total of 192 stool samples were collected from adult population in Cubal, Angola, and examined by parasitological methods (direct saline microscopy and Baermann’s technique). DNA was extracted from each stool sample using a commercial kit and tested by the colorimetric *Strong*-LAMP assay for the detection of *Strongyloides* spp. under field conditions. Furthermore, all samples were shipped to a well-equipped laboratory in Spain, reanalysed by the same procedure and compared with a qPCR method. The overall results after testing were compared.

**Results:**

*Strongyloides stercoralis* larvae were identified by direct saline microscopy and Baermann in a total of 10/192 (5.2%) and 18/192 (9.4%) stool samples, respectively. Other helminth and protozoan species were also identified. The *Strong*-LAMP-positive results were visually detected in 69/192 (35.9%) stool samples. The comparison of Strong-LAMP results in field conditions and at a reference laboratory matched in a total of 146/192 (76.0%) samples. A total of 24/192 (12.5%) stool samples tested positive by qPCR.

**Conclusions:**

This is the first study in which colorimetric Strong-LAMP has been clinically evaluated in a resource-poor strongyloidiasis endemic area. Strong-LAMP has been shown to be more effective in screening for strongyloidiasis than parasitological methods under field conditions and qPCR in the laboratory. Our Strong-LAMP has proven to be a field-friendly and highly accurate molecular test for the diagnosis of strongyloidiasis.

**Graphical Abstract:**

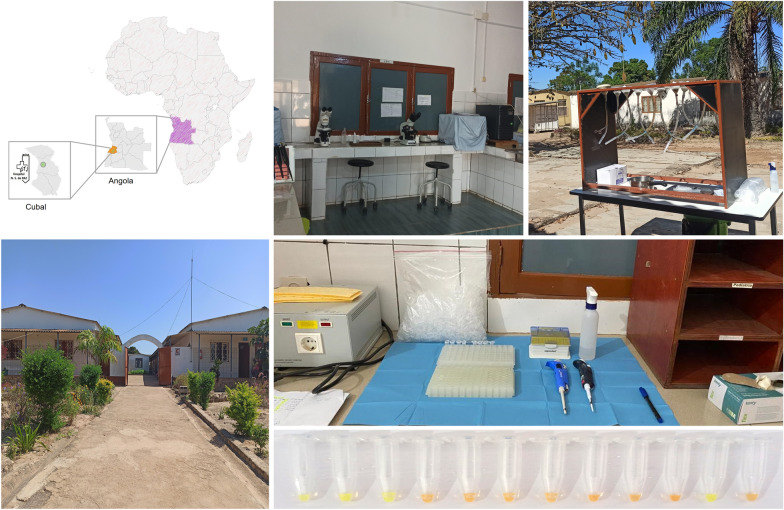

## Background

Strongyloidiasis is a parasitic disease caused by *Strongyloides stercoralis*, a microscopic nematode (roundworm) endemic in tropical and subtropical climates, although foci of infection occur in temperature regions as well. To a lesser extent, *Strongyloides fuelleborni fuelleborni* (in Africa) and *S. fuelleborni kelley* (in Papua New Guinea) are also known to infect humans [1, 2]. Strongyloidiasis is considered the most neglected soil-transmitted helminthiasis (STHs) within the neglected tropical diseases (NTDs). Although real prevalence data in endemic areas are unknown, a recent literature review estimated that about 613.9 million people were infected with *S. stercoralis* in 2017 [3]. More than 76% of the global burden of strongyloidiasis occurs in sub-Saharan Africa, Southeast Asia, Latin America and Western Pacific regions [4].

Strongyloidiasis is acquired primarily by the infective filariform larvae penetrating the skin or mucous membranes though unprotected contact with contaminated soil. Larvae may undergo three separate developmental pathways: internal auto-infective cycle, external direct or external indirect cycle [5]. Uncomplicated strongyloidiasis ranges from asymptomatic or mildly symptomatic to chronic infection for years or decades. However, uncontrolled multiplication of the parasites (hyperinfection syndrome) in immunosuppressed patients (transplant recipients and those on corticosteroid treatment) can be life-threatening with high mortality reaching 85–10% [6, 7].

Strongyloidiasis is one of the most difficult parasitic diseases to diagnose because of three principal concurrent factors: the presence of subclinical or mildly symptomatic cases, the usually low parasite burden and irregular larvae output, and the lack of a gold diagnostic test [8]. The microscopic examination of larvae in faecal samples has insufficient sensitivity [9, 10]. The serological tests have demonstrated high sensitivity but lack specificity because of possible cross-reactions with other parasites and long-term persistence of antibodies after treatment [11]. The molecular methods (mostly based on PCR) have been implemented with the aim to achieve the highest sensitivity while preserving high specificity, but there are discrepancies in their accuracy [12]. Additionally, PCR-based methods are difficult to apply in resource-limited settings because they are technically complex, time-consuming and require skilled personnel and expensive equipment [13]. Therefore, the development of new diagnostic methods for strongyloidiasis that combine features such as low cost, rapidity, simplicity of handling and interpretation and detection capability with high sensitivity and specificity are vital to address the current limitations in the use of PCR-based tests in low-income countries. An alternative could be the loop-mediated isothermal amplification (LAMP) assay, a simple tube method for nucleic acid amplification under isothermal conditions with high sensitivity and specificity [14], allowing the naked-eye discrimination of positive results [15]. At present, LAMP technology brings together all the necessary features of a highly efficient diagnostic assay, together with simple operation, for potential use in the clinical diagnosis of infectious diseases, including point-of-care (POC) testing under field conditions in developing countries [16–18]. A number of LAMP assays have been developed for most parasite-caused NTDs as an alternative molecular tool to PCR-based methods, but to date only a few of them have been tested in real field conditions [19].

To date, for strongyloidiasis, only two LAMP assays have been adapted to detect *Strongyloides* spp. DNA in different types of samples. The first LAMP assay to detect *S. stercoralis* was based on the 28S rRNA gene using *Strongyloides ratti* as a source to determine analytical sensitivity and potential use for diagnosis of human infection in stool samples [20]. Later, this LAMP was compared with qPCR in stool, serum and bronchoalveolar lavage fluid human sample analysis, with fewer LAMP-positive results [21]. On the other hand, our group developed a LAMP method (named *Strong*-LAMP) based on the 18S rRNA gene using *Strongyloides venezuelensis* as a source for the molecular detection of *Strongyloides* spp. DNA in stool and urine samples in a rat infection model. Furthermore, the potential clinical applicability of the *Strong*-LAMP could be demonstrated in several human stool samples with strongyloidiasis confirmed both parasitologically and by qPCR [22]. More recently, the *Strong*-LAMP method demonstrated its efficacy in the detection of *S. stercoralis* DNA in urine samples from patients with confirmed strongyloidiasis and/or the serological suspicion of infection by the parasite [23]. However, these are all laboratory evaluations so it would be very interesting to evaluate the *Strong*-LAMP for the diagnosis of strongyloidiasis in a poor-resource endemic setting under real field conditions.

A recent meta-analysis has showed that reported data from several African countries present a very heterogeneous *S. stercoralis* infection rate that is difficult to compare because of the use of different diagnostic methods, study settings and the characteristics of the population studied [24]. The review concluded that strongyloidiasis prevalence in Africa is overlooked and its prevalence is low because of the use of low-sensitivity diagnostic methods, encouraging the combination of microscopic and molecular-based diagnostic methods to increase sensitivity and establish the true prevalence of strongyloidiasis. In Angola, the current epidemiological information about *S. stercoralis* is still scarce. A limited number of microscopy-based studies in Cubal, a rural area in South Angola, focusing on school-age children reported a strongyloidiasis prevalence of 0.07% [25] and 12.2% [26]. A more recent study determined a considerably higher prevalence rate of 21.4% in children when samples were analysed by qPCR in a reference laboratory [27]. To date, no study in Angola has evaluated the prevalence of strongyloidiasis in the adult population or used a molecular method for diagnosis in surveys under field conditions.

Thus, the aim of this study was to evaluate, for the first time, our previously developed *Strong*-LAMP assay as an easy-to-perform colorimetric molecular method for the detection of *S. stercoralis* DNA in adult stool samples under field conditions in a low-income strongyloidiasis-endemic area in Cubal, Angola, Africa. Furthermore, we evaluated the reproducibility of the *Strong*-LAMP assay in a well-equipped reference laboratory and to compare the results with a qPCR assay commonly used for the diagnosis of strongyloidiasis.

## Methods

### Study area, population and human stool sample collection

The study was conducted between May and August 2022 in the district of Cubal, Benguela Province, western-central Angola, Africa. The municipality of Cubal is formed of Cubal Sede and three urban communes, namely Yambala, Capupa and Tumbulo, with an estimated population of 322,000 inhabitants, where nearly half (151,000; 47%) are children between 5 and 14 years old. Angola lies between and within two major terrestrial biogeographic regions: the moist forests and savannas of the Congolian region and the woodlands, savannas and floodplains of the Zambezian region. These two major divisions occupy over 97% of Angola. In the interior highlands the climate is mild, with a rainy season from November to April, followed by a dry season from May to October [28].

Adults were invited to participate in the study, including inpatients at Nossa Senhora da Paz Hospital (Cubal Sede), outpatients attending the clinical consultation and randomly recruited people in the vicinity of the hospital. For each enrolled participant sociodemographic (age, gender, commune of residence) and clinical (intestinal, respiratory and cutaneous symptoms) information was recorded. After the interview, each participant was given a prelabelled plastic flask for stool sample collection. The collected samples were delivered to the Microbiology Laboratory of the Nossa Senhora da Paz Hospital for processing. A total of 200 participants were initially recruited, but eight had to be excluded because of insufficient stool amount. Thus, a total of 192 participants, including 124 females (64.6%) and 68 males (35.4%), were finally included and tested in the study. A single stool sample was individually obtained from each participant.

### Parasitological analysis

All the stool samples were examined by direct saline microscopy (DSM) and Baermann concentration technique by two specialized personnel. Microscopic detection of *S. stercoralis* larvae (and/or other intestinal parasites) was performed through direct examination with a small portion of each stool sample mixed with saline solution. Another small portion was reserved for further DNA extraction for molecular analysis. To perform the Baermann technique for diagnostic of parasitic larval forms, the rest of the stool sample was mixed with warm water. The loose or watery faeces were first mixed with vermiculite for better processing. Each of the faecal mixtures was then placed on a layer of gauze and placed in a funnel clamped to a rubber tube. The funnel was filled with warm water and incubated under sunlight. After 45 min, the contents of the rubber tube were centrifuged and the sediment was observed under a microscope for the presence of *S. stercoralis* and/or other parasites.

### DNA extraction from stool samples

Approximately 250–300 mg from each of the 192 stool samples was used for DNA extraction using the NZY Soil gDNA Isolation Kit (NZYTECH, Lisbon, Portugal) following the manufacturers’ instructions. Eluted and purified DNA (50 µl) was aliquoted in two vials of the same volume and stored at − 20 ºC until use. Once DNA samples were used for molecular field analysis by LAMP at Nossa Senhora da Paz Hospital, they were stored again at − 20 ºC until they were later shipped to Centre for Research in Tropical Diseases of the University of Salamanca (CIETUS, Salamanca, Spain) for molecular reanalysis by LAMP and qPCR. DNA was extracted and stored as stool samples were collected, so that the first DNA samples obtained were stored for longer (approximately 3 months) than those obtained at the end of the study (approximately 3 weeks) before being shipped to our laboratory in Spain. The DNA samples were kept frozen whenever possible because of possible power outages at the Nossa Senhora da Paz Hospital. Besides, it was not possible to maintain the cold chain during the entire transport of the samples to Spain.

### *Strong*-LAMP: field and reference lab tests

Detection of *S. stercoralis* DNA in stool samples in both field context in Angola and the reference laboratory in Spain was achieved by the colorimetric *Strong*-LAMP assay following the same procedure previously described by Fernández-Soto et al. [22]. A set of four primers based on a 329 base pair sequence *Strongyloides* spp.-derived partial sequence in the 18S rRNA gene was used.

Briefly, the reaction mixtures (15 µl) contained 40 pmol each of FIP and BIP primers, 5 pmol each of F3 and B3 primers, 1.4 mM each of dNTP (Bioron, GmBH, Römerberg, Germany), 1 × Isothermal Amplification Buffer − 20 mM Tris-HCL (pH 8.8), 50 mM KCL, 10 mM (NH_4_)_2_SO_4_, 2 mM MgSO_4_, 0.1% Tween20 (New England Biolabs Ltd., Ipswich, UK)–6 mM supplementary MgSO_4_ and 8 U of *Bst* 2.0 WarmStart DNA polymerase (New England Biolabs Ltd., Ipswich, UK) with 2 µl template purified DNA. Reactions were incubated for 60 min at 65 °C in a heating block and heated at 80 °C for 5–10 min to stop the reaction. Amplification assays were performed in batches of 10 samples each for easy handing and avoid cross-contamination. Each batch always included several negative (water) and positive (DNA from *S. venezuelensis* infective filiform larvae, L3; 5 ng/µl) controls. After the reaction was finished, 2 µl of 1:10 diluted 10,000 concentration SYBR^®^ Green I dye was carefully added to the reaction tubes for the naked-eye visualization of the results by colour change (green: positive; orange: negative).

Once at our reference laboratory at CIETUS (Salamanca, Spain), all 192 DNA samples were reanalysed by the colorimetric *Strong*-LAMP by the same procedure used in the endemic area (Angola) as mentioned above to evaluate the reproducibility of the technique. All LAMP tests carried out in the field and at the reference laboratory were performed and tested by the same experienced researcher.

### qPCR assay for *S. stercoralis* detection

The detection of *S. stercoralis* DNA was also achieved at the reference laboratory by a qPCR assay targeting the small subunit ribosomal RNA (*SSU* rRNA) gene of *Strongyloides* spp. as first described elsewhere by Verweij et al. [29]. In our study, qPCR reactions were performed in a final volume of 20 µl containing the commercial NZYSupreme qPCR Green Master Mix (2x) (NZYTECH, Lisbon, Portugal), 0.2 µM of each specific primer (Eurofins Genomics, GmbH, Ebersberg, Germany) and 2 µl template DNA. Genomic DNA from *S. venezuelensis* L3 and ultrapure water was included as positive and negative control, respectively, in each run. The amplification program was carried out in the Eco 48 Real Time qPCR System (PCR max) device and consisted of an initial polymerase activation step at 95 °C for 3 min, followed by 35 cycles of 95 °C for 5 s for denaturation and 60 °C for 25 s for annealing/extension. DNA samples were tested in duplicate.

### Statistical analysis

To estimate the accuracy of the *Strong*-LAMP assay method as a diagnostic test, the percentages of the sensitivity, specificity, positive predictive value (PPV) and negative predictive value (NPV) were calculated in comparison to both DSM and Baermann using the WinEpi 2.0 statistical free software [30]. The confidence intervals (CI) were established at 95%. McNemar’s statistical test was used to compare the diagnostic methods (*Strong*-LAMP against microscopy and Baermann technique). A Cohen’s kappa coefficient was also performed to evaluate the concordande between *Strong*-LAMP under field conditions and at a well-equipped laboratory.

## Results

### Parasitological analysis

By DSM examination *S. stercoralis* larvae were identified as a single agent in a total of 10/192 (5.2%) human stool samples. Other intestinal parasites were identified in a total of 32/192 (16.7%) individuals, including helminths (17/192; 8.9%) and protozoa (15/192; 7.8%). The species identified and their corresponding percentages are listed in Table 1. Additionally, 5/192 (2.6%) participants presented a coinfection with two parasites, including: *Ascaris lumbricoides*/*S. stercoralis*, *Giardia lamblia*/*S. stercoralis*, hookworm/ *Balantidium coli*, hookworm/*Ascaris lumbricoides* and *A. lumbricoides*/*S. stercoralis*.

**Table 1:** Prevalence (%) of helminth and protozoan species identified by DSM and Baermann in stool samples from the 192 included in this study

Identified parasites	DSM	Baermann	Total
Helminths			
*Strongyloides stercoralis*	10 (5.2%)	18 (9.38%)	18 (9.4%)
Hookworm	7 (3.7%)		7 (3.7%)
*Ascaris lumbricoides*	4 (2.1%)		4 (2.1%)
*Taenia* spp.	3 (1.6%)		3 (1.6%)
*Trichuris trichiura*	1 (0.5%)		1 (0.5%)
*Hymenolepis* spp.	1 (0.5%)		1 (0.5%)
*Schistosoma mansoni*	1 (0.5%)		1 (0.5%)
Protozoa			
*Giardia lamblia*	12 (6.3%)		12 (6.3%)
*Balantidium coli*	1 (0.5%)	3 (1.6%)	4 (2.1%)
*Entamoeba histolytica/dispar*	2 (1.0%)		2 (1.0%)

By Baermann technique, *S. stercoralis* larvae were identified in a total of 18/192 (9.4%) individuals, including those 10 DSM-positive samples, thus increasing the parasitological positive results by eight. Additionally, *B. coli* was the only species detected by Baermann technique in three more samples compared to microscopy examination (Table 1).

### *Strong*-LAMP analysis under field conditions

**Fig. 1:**
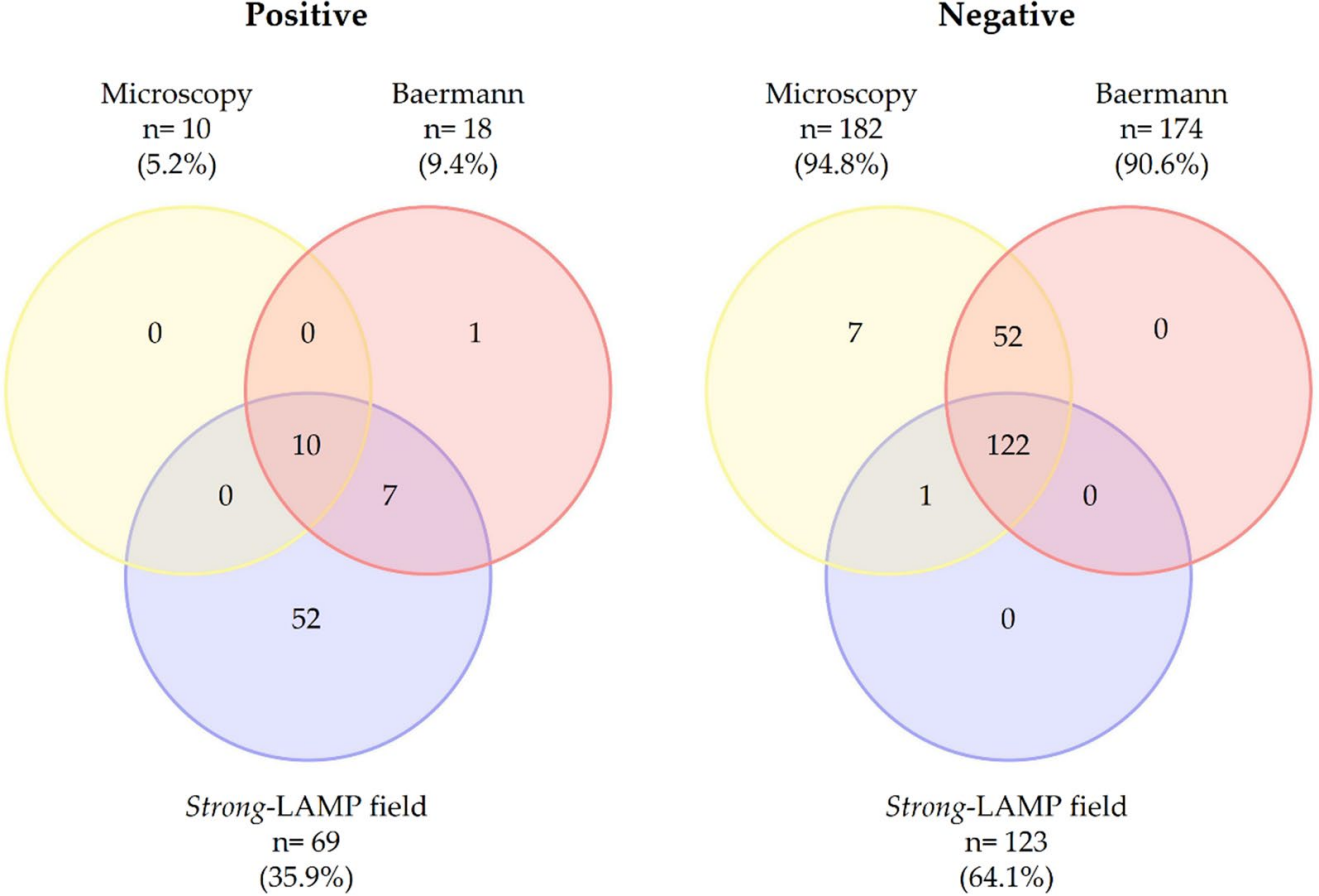
Overall results obtained by *Strong*-LAMP assay of human stool samples (*n* = 192) under field conditions compared with direct saline microscopy (DSM) and Baermann’s method for *Strongyloides stercoralis* detection. Venn diagrams for three-way comparison of *Strong*-LAMP, microscopy and Baermann showing overlapping of number of positive (left) and negative (right) results and percentages obtained

The overall results of *Strong*-LAMP obtained after testing purified DNA from stool samples under field conditions compared to microscopy and Baermann’s method findings are summarised using Venn diagrams in Fig. 1. The *Strong*-LAMP-positive results were visually detected by green colour in a total of 69/192 (35.9%) stool samples; 123/192 (64.1%) remained orange (negative).

All DSM-positive samples (10/192) were both Baermann and *Strong*-LAMP positive. In addition, up to 17 of the 18 samples with a positive result by Baermann also were *Strong*-LAMP positive. Only one sample was Baermann positive and up to 10 samples were positive by the three techniques. On the other hand, 122 stool samples were negative for all three techniques applied.

For the *Strong*-LAMP assay in this study, the sensitivity, specificity, PPV and NPV diagnostic parameters were calculated considering the parasitological techniques, either DSM or Baermann, as the reference standard method in endemic area. The results are presented in Table 2. McNemar’s test showed a statistically significant relation between *Strong*-LAMP results and DSM-positive results (χ^2^ = 57.017, *df* = 1, *P* < 0.001) as well as between *Strong*-LAMP results and Baermann technique results (χ^2^ = 6.12, *df* = 1, *P* < 0.01). A 44.8% (kappa 0.44) concordance between *Strong*-LAMP under field conditions and at a reference laboratory was obtained.

**Table 2:** Sensitivity, specificity, positive predictive value (PPV), negative predictive value (NPV) and likelihood ratios of *Strong*-LAMP versus microscopic diagnostic methods

Diagnostic test	Sensitivity (95% CI)	Specificity (95% CI)	PPV (95% CI)	NPV (95% CI)
*Strong*-LAMP/DSM	100% (100–100%)	67.60% (60.8–74.4%)	14.5% (6.2–22.8%)	100% (100–100%)
*Strong*-LAMP/Baermann	94% (83.9–105.0%)	70.10% (63.3–76.9%)	24.60% (14.5–34.8%)	99.20% (97.6–100.8%)

*DSM*, direct saline microscopy

### *Strong*-LAMP assay at a reference laboratory

**Fig. 2:**
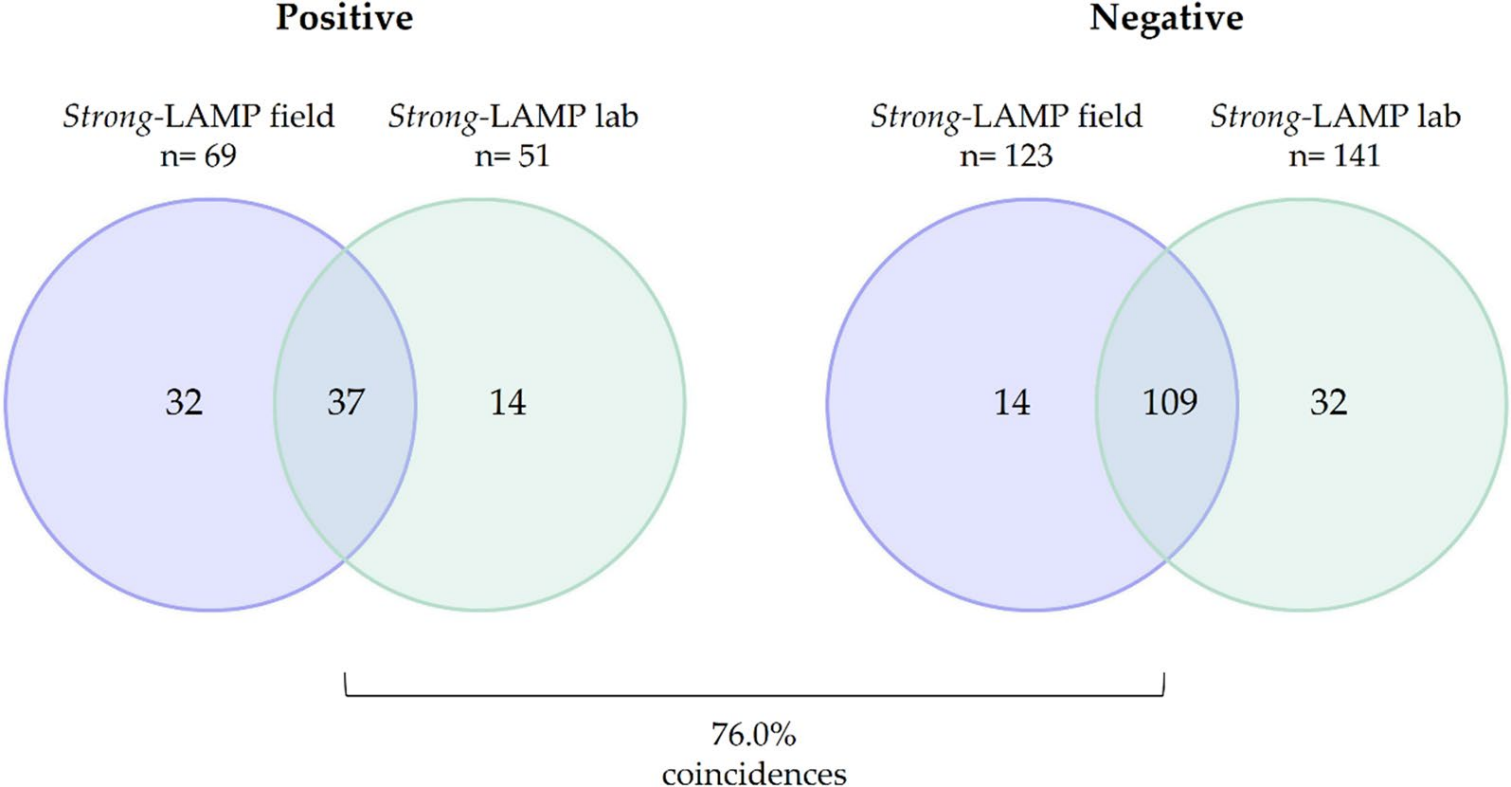
Comparison of *Strong*-LAMP results under field conditions and at a reference laboratory. Positive (left), Venn diagrams showing coincidences of positive results. Negative (right), Venn diagrams showing coincidences of negative results

Comparison of the *Strong*-LAMP results obtained at a reference laboratory with those obtained under field conditions is shown in Fig. 2. In our laboratory, 51/192 (26.6%) were *Strong*-LAMP positive. Overall results matched in a total of 146/192 (76.0%) samples, counting 37/69 (53.6%) for positive and 109/123 (88.6%) for negative results.

At laboratory, *Strong*-LAMP amplified all DSM-positive samples and up to 16 out of 18 Baermann-positive samples under field conditions. Of the two Baermann-positive samples that were negative, one had also been negative for *Strong*-LAMP under field conditions.

### qPCR at reference laboratory

qPCR-positive results are summarized in Table 3. A total of 24/192 (12.5%) stool samples tested positive and 168/192 (87.5%) tested negative by qPCR, providing cycle threshold (Ct) values within the range of 23.5–35.7 (median: 31.6). No amplification was detected in 6/18 samples (nos. 52, 159, 32, 57, 70 and 149) that tested positive by Baermann, including 2 samples (nos. 52 and 159) that were also positive by DSM. Note that two qPCR-positive results were obtained for two samples (nos. 25 and 79) with microscopy positive for hookworms but negative for *S. stercoralis*.

**Table 3:** Positive qPCR results obtained in the analysis of the stool samples included in the study indicating the corresponding cycle threshold

Sample no.	DSM	Baermann	*Strong*-LAMP field	*Strong*-LAMP lab	qPCR	Ct
10	*Strongyloides stercoralis* (3L)	*S. stercoralis* (25L)	+	+	+	28.7
15	–	–	–	+	+	33.8
16	–	–	–	+	+	23.5
21	–	–	+	+	+	33.6
25	Hookworm	–	–	–	+	29.5
38	*S. stercoralis* (1L)	*S. stercoralis* (5L)	+	+	+	29.3
46	–	*S. stercoralis* (1L)	+	+	+	35.7
59	–	*S. stercoralis* (2L)	+	+	+	34.7
60	–	–	–	+	+	33.5
61	–	–	–	+	+	34.7
67	*S. stercoralis* (1L)	*S. stercoralis* (1L)	+	+	+	28.6
71	–	–	+	+	+	33.7
73	*Ascaris lumbricoides*	*S. stercoralis* (3L)	+	+	+	33.7
78	*S. stercoralis* (1L)	*S. stercoralis* (4L)	+	+	+	28.9
79	Hookworm	–	+	–	+	32.7
82	–	–	+	+	+	32.3
83	*S. stercoralis* (1L)	*S. stercoralis* (4L)	+	+	+	28.4
108	*S. stercoralis* (1L)	*S. stercoralis* (1L)	+	+	+	26.0
112	*S. stercoralis* (43L)	*S. stercoralis* (63L)	+	+	+	33.4
114	–	–	–	+	+	33.9
122	*Giardia/S. stercoralis* (1L)	*S. stercoralis* (2L)	+	+	+	29.3
126	–	–	+	+	+	34.5
168	–	–	–	–	+	33.3
192	–	*S. stercoralis* (1L)	+	+	+	31.8
52	*S. stercoralis* (1L)	*S. stercoralis* (2L)	+	+	–	
159	*S. stercoralis* (1L)	*S. stercoralis* (1L)	+	+	–	
32	–	*S. stercoralis* (1L)	+	+	–	
57	–	*S. stercoralis* (1L)	+	–	–	
70	–	*S. stercoralis* (1L)	+	+	–	
149	*A. lumbricoides*/Hookworm	*S. stercoralis* (1L)	–	–	–	
Total +			22	25	24	

*DSM* Direct saline microscopy, *Ct* Cycle threshold. Negative qPCR results in those samples with positive parasitological results in field conditions (DSM and Baermann) and comparison with the results obtained both in *Strong*-LAMP in the field and at a reference laboratory are also included. In samples with a positive parasitological result for *S. stercoralis*, the number of larvae counted in the microscopic preparation examined is indicated in parentheses.

Comparison of the qPCR results with *Strong*-LAMP assays, both under field conditions and at a reference laboratory, is shown in Table 4. The coincidences of the results between qPCR and *Strong*-LAMP under field conditions accounted for 69.3%, while the coincidences of qPCR and *Strong*-LAMP at laboratory were 82.8%.

**Table 4:** Comparison of the qPCR results with *Strong*-LAMP assays, both under field conditions and at a reference laboratory

	Positive/negative	qPCR		Coincidences
		24 Positive	168 Negative	
*Strong*-LAMP (field)	69 Positive/123 negative	17	116	133/192 (69.3%)
*Strong*-LAMP (lab)	51 Positive/141 negative	21	138	159/192 (82.8%)

**Fig. 3:**
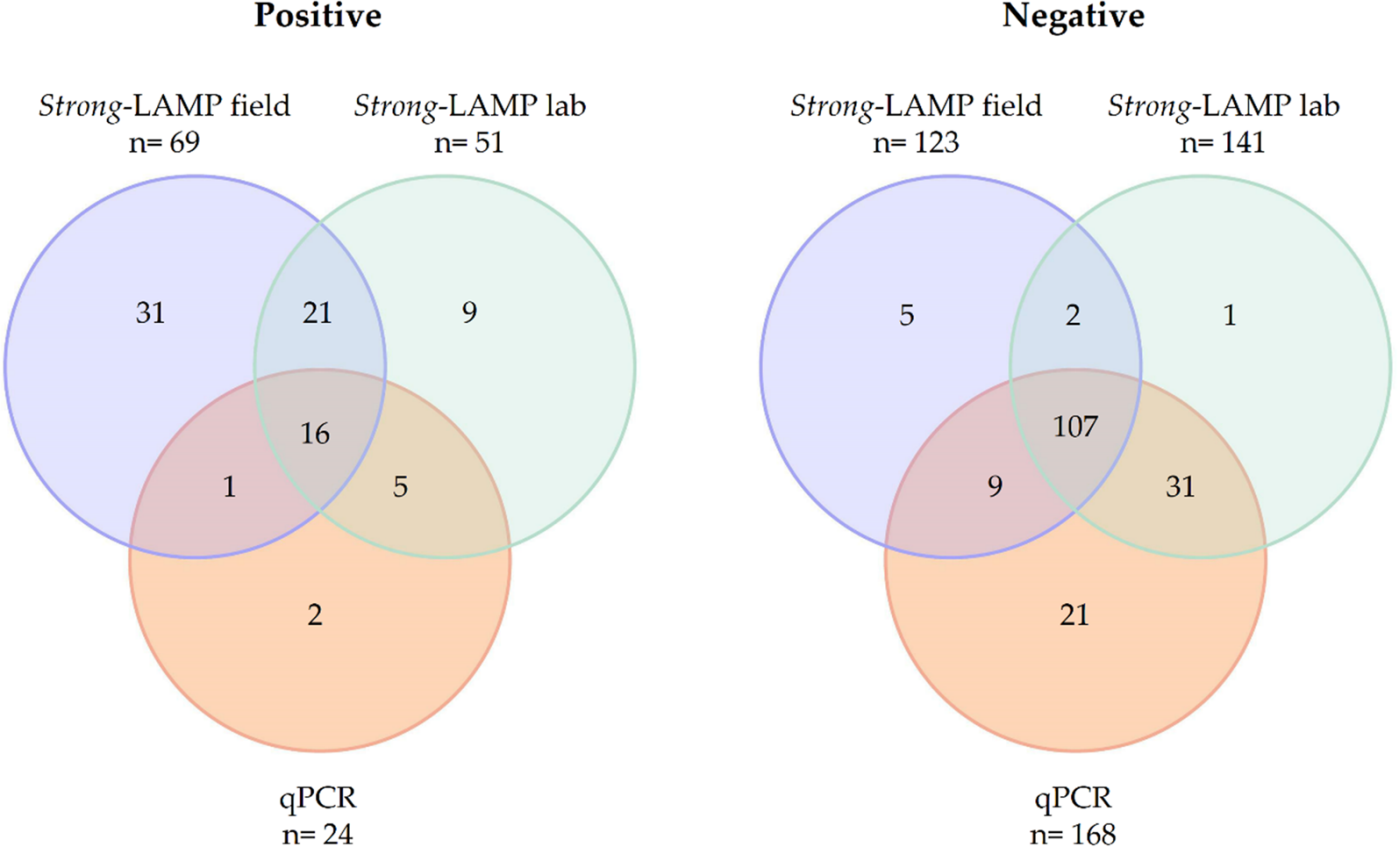
Overall results obtained by qPCR method compared with *Strong*-LAMP under field conditions (field) and *Strong*-LAMP at a reference laboratory (lab) for *Strongyloides stercoralis* detection. Positive (left), Venn diagrams for three-way comparison of positive results. Negative (right), Venn diagrams for three-way comparison of negative results

A three-way comparison of the results of qPCR, *Strong*-LAMP under field conditions and *Strong*-LAMP at laboratory results is shown in Venn diagrams in Fig. 3. Up to 16 samples and 107 samples were positive and negative, respectively, for the three molecular assays performed.

## Discussion

Recently, the World Health Organization (WHO) introduced the activities and targets on the road to control of strongyloidiasis by 2030, and the identification of a test that could be deployed in the field and the results documented is urgent and imperative [31]. The WHO already recognized the lack of standardized diagnostic methods and held a virtual meeting on ‘Diagnostic methods for the control of strongyloidiasis’ on September 29, 2020 [32]. During this meeting, the suitability of the currently available diagnostic methods (coprological, serological and PCR-based) to estimate the prevalence of the disease at population level was evaluated. Different perspectives and analyses of each category of diagnostic tests were presented and, in an overview of molecular diagnostics, the LAMP assays developed to date by Watts et al. [20] and by Fernández-Soto et al. [22] for the detection of *S. stercoralis* were also discussed. Their good analytical sensitivity and promising diagnostic use were highlighted, as was the limitation of not yet having been more widely clinically validated.

In this work, we tested our colorimetric *Strong*-LAMP assay [22] for *S. stercoralis* DNA detection in a total of 192 human stool samples compared to both DSM and Baermann technique in a low-income strongyloidiasis-endemic area in Cubal, Angola. The reproducibility of the *Strong*-LAMP assay was also later evaluated in a reference laboratory and compared with a qPCR method.

The prevalence values of *S. stercoralis* infection were 5.2% and 9.3% as observed using DSM of faecal smears and Baermann’s technique, respectively. The better sensitivity of Baermann obtained compared to DSM is not surprising as it is well known and has been evidenced in many different studies [10]. The pooled parasitological prevalence value in our study (9.3%) was slightly lower than the infection rate (12.2%) previously reported in an Angolan children population by using formol ether concentration technique (FECT) and Baermann [26] but higher than 0.07% also reported in school-children population of Cubal, Angola, by only FECT [25]. In general, our data are higher than the pooled low prevalence of strongyloidiasis reported by different African countries across different parasitological diagnostic methods (mainly DSM, FECT and Baermann) and study settings (schools, health institutions and rural communities) in a recent review by Hailu et al. [24]. Notwithstanding, we are aware that the analysis of a single faecal sample per patient, the intermittent shedding of *S. stercoralis* larvae in faeces, and a low larval load in low-incidence infections may have affected the estimation of a true prevalence by simple parasitological diagnosis [2, 9]. Indeed, a combination of microscopic and molecular-based diagnostic methods (when possible) to determine the true prevalence of strongyloidiasis has been recommended [24, 33]. Thus, to increase the sensitivity of *S. stercoralis* detection, the convenience and simplicity of a colorimetric LAMP assay in the field would be a great advantage over other conventional molecular methods and could easily be used as a complementary tool for the diagnosis of strongyloidiasis. In this regard, when we tested the stool samples by *Strong*-LAMP, the prevalence of human strongyloidiasis increased to 35.9%. The absence of information in already published data on the LAMP-based prevalence for *S. stercoralis* under field settings does not allow us to compare our results. However, what we can highlight is that the prevalence value of strongyloidiasis found by *Strong*-LAMP in the present study is considerably higher than those available for other African countries (ranging 1.1% to 21.9%; with the exception of Mozambique: 48.5%) when PCR is used as a molecular diagnostic method [24]. Statistically, compared to DSM and Baermann, the *Strong*-LAMP showed the highest sensitivity (100% and 94.4%, respectively), although PPV was low (14.5% and 24.6%, respectively). A high sensitivity value and a low PPV are in line with our experience working with different LAMP assays when comparing their accuracy with parasitological techniques used as reference in diagnosis with different types of clinical samples, including urine (for urinary schistosomiasis) [34], faeces (for amphimeriasis) [35] or blood (for loiasis) [36].

To compare different LAMP application scenarios, the performance of the *Strong*-LAMP colorimetric assay under field conditions in Angola was reassessed in our laboratory in Spain. The overall 35.9% prevalence obtained in field decreased to 26.6% in the laboratory. One of the possible causes could be the inadequate storage of the purified DNA samples under field conditions, as they underwent repeated freeze-thaw cycles due to power outages in the area during the study. Furthermore, it was not possible to maintain the cold chain for shipment to our laboratory, thus increasing the potential degradation of DNA. In addition to other possible factors, it has been shown that freeze-and-thaw cycles cause degradation of standard DNA, thus decreasing the amplification by molecular methods, especially for real-time PCR [37]. The decreased efficacy of LAMP in the laboratory compared to the field had already been observed in a previous study when we analysed DNA from clinical urine samples for *Schistosoma haematobium* after inadequate cold chain maintenance for preservation and subsequent shipment of the samples [34]. Considering that, in many cases, a lack of reagents, equipment or time may exist, necessary at the place and time where the survey is conducted, we emphasize the importance of proper preservation and shipment of samples if their subsequent analysis in appropriately equipped laboratories is required. At this point, our work is subject to the limitation that no DNA stabilizers or other sample preservation methods could be used. In this regard, common filter paper might be useful for easy collection and long-term storage of human stool samples in field settings for subsequent DNA extraction and molecular analysis for *S. stercoralis* in a refence laboratory. This methodology has already given us good results in a previous field study for stool sample collection and subsequent extraction and detection of parasite DNA by LAMP in the laboratory [35]. Further research is needed to explore other DNA collection, preservation, shipment and extraction systems that will allow standardization in the detection of *S. stercoralis* DNA.

Nevertheless, it should be noted here that regardless of the quality of the DNA used, *Strong*-LAMP-positive results obtained under field conditions and subsequently in our reference laboratory were consistent for all samples with a DSM-positive finding (10/10) and almost all Baermann findings (16/18; note that of the 2 Baermann-positive samples that were negative, one had also been negative for *Strong*-LAMP under field conditions) meaning that *Strong*-LAMP detected true *S. stercoralis* infections. This outcome reinforces the reliability of our colorimetric *Strong*-LAMP assay for *S. stercoralis* DNA detection in human stool samples, both under field conditions and in a potential clinical setting.

On the other hand, a widely used qPCR first described by Verweij et al. [29] was also tested as a complementary molecular technique because this qPCR was previously used in Cubal (Western Angola) for the detection of *S. stercoralis* DNA in stool samples collected from children [27]. In the present study, the detection rate of *S. stercoralis* by qPCR was lower (24/192; 12.5%) than that of *Strong*-LAMP under field conditions (69/192; 35.9%) and in laboratory (51/192; 26.6%). This qPCR-based prevalence for *S. stercoralis* was also substantially lower than that previously reported in Cubal in school children (21.4%) [27]. Also using this qPCR, a prevalence of *S. stercoralis* of 13.4% [38] and, more recently, a prevalence of 28.8% [39] has been reported in the Ethiopian child population. On the other hand, significantly different prevalence rates of *S. stercoralis* of 1.1% in Ghana [40], 7.4% in Tanzania [41] and up to 48% in Mozambique [42] have been found when testing in general population. Although it has been suggested that the increase in prevalence in the Mozambican population could be age related, probably as a result of autoinfection and chronic infection [27], what is of relevance is that there are many discrepancies in the reported accuracy of PCR-based tests. Those discrepancies can be attributed to numerous variables, such as the setting in which the research was conducted, population, comparator, etc. In fact, it has been highlighted that PCR-based methods for *S. stercoralis* might not be suitable for screening purposes, whereas they might have a role as a confirmatory test, as it still misses a relevant proportion of infected people [12]. Additionally, the reproducibility of PCR-based tests for *S. stercoralis* detection in different laboratories has not yet been evaluated.

Surprisingly, two qPCR-positive results (both with a negative *Strong*-LAMP result in the laboratory test, although one positive in field test) were obtained for two samples with positive microscopy for hookworms. Considering that no cross-reactions with hookworms have been described for either qPCR [29] or *Strong*-LAMP [22], it is likely that these two samples were indeed positive for *S. stercoralis* and would not have been detected by the less sensitive parasitological methods.

Notably, in our study, qPCR did not amplify two DSM-positive samples and up to six Baermann-positive samples that did by *Strong*-LAMP, in both the field and the laboratory. We can speculate that besides the inadequate DNA storage, this could have been due to a possible inhibition of qPCR. Substances typically present in human faeces and dietary components can limit DNA extraction success [43]. Also, complexity of stool samples contain substances and subsamples that can inhibit PCR and may lead to decreased PCR sensitivity or even false-negative PCR results [44, 45]. Moreover, the same inhibitory substances might not always be equally inhibitory for all PCR reactions or initial sample types [46]. In this sense, several reports have demonstrated that LAMP technology is more robust and tolerates higher levels of sample-derived inhibitors from biological samples than PCR [47–49]. With this in mind, it is plausible that our *Strong*-LAMP was able to detect all parasitological positive samples for *S. stercoralis* but not qPCR and, overall, a higher number of positive samples than qPCR throughout the study.

We should also emphasize here that LAMP offers a number of well-known advantages over PCR-based methods in field settings, such us minimal equipment, short time to result (30–45 min) and similar or higher sensitivity to PCR, and results can be simply read by the naked eye using a dye [14, 15, 50, 51]. Most African countries use low-sensitivity parasitological diagnostic methods for the detection of *S. stercoralis* because the more sensitive molecular methods are expensive and difficult to adopt in most African health institutions [24]. Therefore, as highlighted by Njiru et al. [16], we should direct our attention to LAMP application in poorly equipped and low-resourced laboratories to provide developing countries a rapid, sensitive, specific, reliable and easily available diagnostic instruments. As early as 2006, the acronym ASSURED (Affordable, Sensitive, Specific, User-friendly, Rapid and Robust, Equipment-free, Deliverable) was proposed by the WHO Sexually Transmitted Diseases Diagnostics Initiative as a set of criteria that any diagnostic method must achieve to be considered as a point-of-care (POC) test in low resource settings [52]. This term has been recently updated to (RE)ASSURED, including Real-time connectivity and Ease of specimen collection and Environmental friendliness [53]. A great variety of recent approaches of the LAMP technology in a field-friendly display have been released (e.g. lateral flow dipsticks, microchips, laboratory-on-chips, portable fluorometers or smartphone apps) that may help it to achieve REASSURED criteria, without prejudice to the necessary improvements that still need to be made for use as a true POC [19].

In this line, a recent study by our group has proved that the combination of long-term stabilized LAMP master mix for real-time colorimetric *Strongyloides* spp. DNA detection using our patented smartphone-operated SMART-LAMP handheld device would provide an improvement towards true POC diagnosis of strongyloidiasis in settings with limited infrastructure [54]. More recently, we have also adapted the *Strong*-LAMP to a duplex-LAMP format for the simultaneous detection of strongyloidiasis and schistosomiasis (easily customizable to other coinfections) in a portable real-time platform [55]. Thus, in view of the field results obtained in the present study with the simple colorimetric *Strong*-LAMP and the (RE)al possibility of adaptation to different user-friendly real-time formats, we believe that the *Strong*-LAMP test can be considered as a molecular tool with great potential in the POC diagnosis of strongyloidiasis in endemic areas with limited resources.

## Conclusions

This is the first study in which colorimetric *Strong*-LAMP has been evaluated on human stool samples in a resource-poor strongyloidiasis endemic area in Angola, Africa. *Strong*-LAMP has been shown to have better sensitivity than parasitological tests such as DSM and Baermann’s under field conditions and higher diagnostic sensitivity in the laboratory than the more commonly used qPCR in strongyloidiasis studies. The ease of use and efficacy of the colorimetric assay suggest that *Strong*-LAMP may be a very useful molecular methodology to improve diagnosis of strongyloidiasis, simplifying complex testing of labour-intensive field studies and contributing to improved prevalence surveys. In addition, the real possibility of adapting *Strong*-LAMP (REal) to portable field-friendly devices could soon further increase its value in POC diagnosis of strongyloidiasis.

## Data Availability

All data are contained in the tables and figures.

## Abbreviations

LAMP: Loop-mediated isothermal amplification
qPCR: Quantitative polymerase chain reaction or real-time PCR
PCR: Polymerase chain reaction
STHs: Soil-transmitted helminthiasis
NTDs: Neglected tropical diseases
DSM: Direct saline microscopy
PPV: Positive predictive value
NPV: Negative predictive value
Ct: Cycle threshold
WHO: World Health Organization
FECT: Formol ether concentration technique
ASSURED: Affordable, Sensitive, Specific, User-friendly, Rapid and Robust, Equipment-free, Deliverable
POC: Point-of-care
(RE)ASSURED: Real-time connectivity, ease of specimen collection, environmental friendliness, affordable, sensitive, specific, user-friendly, rapid and robust, equipment-free, deliverable
